# Sodium hexachloroplatinate (IV) induces concentration- and time-dependent depolarization and cytotoxicity in HEI-OC1 auditory cells

**DOI:** 10.1371/journal.pone.0354369

**Published:** 2026-07-22

**Authors:** Hamdy Embark, Kirsten Wissel, Gudrun Brandes, Andrej Kral, Thomas Lenarz, Nils Prenzler, Hannes Maier

**Affiliations:** 1 NIFE, Hannover, Germany; 2 Department of otorhinolaryngology, Hannover Medical School, Hannover, Germany; 3 Institute of neuroanatomy and cell biology, Center of anatomy and cell biology, Hannover Medical School, Hannover, Germany; 4 Institute for AudioNeuroTechnologie (VIANNA), Department of experimental otology, and Cluster of Excellence “Hearing4all.connects”, Hannover Medical School, Hannover, Germany; SVM Arts, Science and Commerce College, INDIA

## Abstract

Over the past decades, cochlear implants (CIs) have been widely acknowledged for their efficacy and reliability in restoring auditory function in patients with severe to profound sensorineural hearing loss. Nonetheless, electrode corrosion involving platinum (Pt) components in CI systems can lead to increased electrode impedance and the release of Pt corrosion products, which possibly have toxic effects on cochlear sensorineural structures. Despite advances in CI technology, the biological responses, mediating cell damage, induced by platinum corrosion products following cochlear implantation remain incompletely characterized. To address this gap, we employed the **H**ouse **E**ar **I**nstitute–**O**rgan of **C**orti **1** (HEI-OC1) cell line as an *in vitro* auditory model to characterize the cytotoxic effects of sodium hexachloroplatinate (IV) (Na_2_[PtCl_6_]) through electrophysiological analyses and relative quantification of mitochondrial oxidative activity. Using whole-cell patch-clamp recordings in current-clamp mode, we observed that Na_2_[PtCl_6_] caused a significant, concentration- and time-dependent depolarization of resting membrane potentials, potentially facilitating activation of voltage-gated ion channels and disrupting ionic homeostasis. Consistent with the intracellular accumulation of Pt and the electrophysiological findings, significant concentration-dependent changes in oxidative activity were observed in HEI-OC1 cells following Na_2_[PtCl_6_] exposure. No time-dependent differences in metabolic activity were detected after 24 h and 48 h of exposure, suggesting that cellular repair mechanisms may be activated in parallel during cultivation. Overall, these findings suggest that this Pt(IV) compound directly alters auditory cell electrophysiology, thereby promoting cytotoxicity. Understanding these mechanisms will improve the development of better implant materials, optimized stimulation pulse protocols, and protective therapies to enhance CI longevity and safety.

## Introduction

Cochlear implants (CIs) have fundamentally transformed the clinical management of severe to profound sensorineural hearing loss by enabling substantial auditory restoration and improving quality of life worldwide [1–3]. The core function of CIs depends on delivering electrical stimuli with electrode arrays implanted into the scala tympani of the cochlea, which directly activate the spiral ganglion and the auditory nerve fibers [4–7]. More than one million CI devices have been implanted globally, demonstrating widespread adoption and effectiveness of this neuroprosthetic approach [8–11]. Despite their reliability, CIs remain prone to functional decline and device failure over time, necessitating ongoing monitoring and possible revision procedures [12–14].

Platinum (Pt) is widely employed as the electrode material in CI devices due to its exceptional biocompatibility and favorable electrical characteristics [15,16]. Nevertheless, extensive evidence demonstrates that prolonged implantation may induce electrochemical corrosion of Pt electrodes, leading to the release of Pt corrosion products—predominantly Pt(II) and Pt(IV) species—into the cochlear microenvironment [17–21]. The formation of these species occurs via oxidation and complexation reactions, which are modulated by local physiological parameters including pH, chloride ion concentration, and electrical stimulation [22–25]. Importantly, dissolved Pt species *in vitro*, generated following electrical stimulation of Pt wires, have been documented to exert cytotoxic effects on cell lines such as the murine fibroblast (NIH 3T3) and neuroblastoma (SH-SY5Y) cell lines [26]. Moreover, cell death induction could be shown in the mouse hair cell line (HEI-OC1) and primary inner ear sensory cells and neurons following Na_2_[PtCl_6_] application for 48 h [27].

Heavy metal-induced cytotoxicity is commonly associated with mitochondrial dysfunction, oxidative stress, and transcriptional dysregulation, which have been widely reported across models of metal and nanoparticle exposure [28–30]. In particular, cadmium-related alterations in oncogenic signaling such as c-Myc regulation, as well as mitochondrial apoptotic responses characterized using targeted probes such as triphenylphosphonium-based systems, illustrate shared mechanisms of metal-induced cellular stress [31,32]. These effects are closely linked to disturbances in ionic homeostasis and membrane integrity, which can converge on signaling pathways including CREB-associated regulation and NF-κB activation observed in chemotherapy-related toxicity models [33,34]. Similar ion imbalance–driven responses have also been reported in nanoparticle exposure systems, including alumina-based models of neurotoxicity [35].

Membrane potential (MP) constitutes a fundamental biophysical property inherent to all living tissues and is indispensable for the physiological function of both excitable and non-excitable cells [36]. Under resting conditions, healthy cells typically maintain a stable resting membrane potential (RMP) ranging approximately from −10 mV to −200 mV, with precise values dependent on cell type and species [37,38]. The RMP is critical for maintaining ion homeostasis and supports key cellular processes, including action potential generation, signal transduction, and regulation of cellular excitability [39]. Deviations from the RMP, particularly sustained depolarization that diminishes membrane negativity, have been shown to impair cellular integrity and disrupt normal physiological functions [40]. Moreover, changes in RMP are often associated with alterations in membrane capacitance, reflecting modifications in cell size, surface area, or membrane properties [41].

This study focused on the impact of sodium hexachloroplatinate (IV) (Na_2_[PtCl_6_]) on the RMP and mitochondrial oxidative activity of the hair cell line HEI-OC1 in a concentration- and time-dependent manner. As described by Kalinec *et al*., the HEI-OC1 cell line, derived from mouse auditory sensory epithelium, served as a valuable *in vitro* cell culture model to study cochlear cell physiology and toxicology [42]. The water soluble and chemically stable Na_2_[PtCl_6_] was chosen as the model Pt(IV) complex, since Rosenberg *et al*. identified an octahedral [PtCl_6_]^2-^ complex which suppressed *Escherichia coli* bacteria growth following electrolysis of Pt electrodes [43,44]. Thus, Na_2_[PtCl_6_] used in this study may represent one of the Pt(IV) compounds potentially generated during Pt corrosion *in vivo*. Whole-cell patch-clamp recordings, together with determination of the mitochondrial oxidative activity, were used to characterize the extent of Pt(IV) toxicity on membrane stability and function.

## Materials and methods

### Preparation of sodium hexachloroplatinate (IV) (Na_2_[PtCl_6_])

A 500 mg/mL stock solution of Na_2_[PtCl_6_] (Sigma–Aldrich, Taufkirchen, Germany) was prepared using sterile double-distilled water. Working solutions were subsequently generated by diluting the stock solution in high-glucose Dulbecco’s Modified Eagle’s Medium (DMEM; Bio Sell, Germany) supplemented with 10% fetal calf serum (FCS; Bio&Sell, Germany), yielding final concentrations ranging from 8 to 12 ng/μL. The concentration range employed in the patch-clamp experiments was selected based on data obtained from our previous study involving HEI-OC1 cells treated with Na_2_[PtCl_6_] [27].

### HEI-OC1 cell cultivation and Na_2_[PtCl_6_] supplementation

HEI-OC1 cells, kindly provided by Prof. Dr. Axel Schambach and Dr. Michael Morgan (Institute of Experimental Hematology, Hannover Medical School, Hannover, Germany), were originally obtained from Dr. Federico Kalinec (House Ear Institute, Los Angeles, CA, USA) and cultured in high-glucose DMEM supplemented with 10% FCS under permissive conditions at 33°C in a humidified atmosphere containing 10% CO_2_. Cells were passaged every 3–4 days using 0.05% trypsin and 0.02% EDTA. As described previously [27], 4,000 cells in 100 µL of DMEM supplemented with 10% FCS were seeded per well in 96-well cell culture plates (TPP, Trasadingen, Switzerland). After pre-cultivation for 24 h at 33°C and 10% CO_2_, the culture medium of experimental wells was replaced with medium containing 8, 10, or 12 ng/µL Na_2_[PtCl_6_], while control wells received fresh medium without Pt. Cells were further cultivated for 24 h or 48 h. Statistical evaluation was performed on three independent experiments (N = 3), with each assay conducted in triplicate (n = 3). Untreated HEI-OC1 cells served as reference for relative quantification of Na_2_[PtCl_6_]-treated samples.

### Resazurin assay for examination of the oxidative activity of HEI-OC1 cells after exposure to Na_2_[PtCl_6_]

The relative quantification of the metabolic activity of the HEI-OC1 cells exposed to varying concentrations of Na_2_[PtCl_6_] was performed by using the VisionBlue™ Quick Cell Viability Fluorometric Assay Kit (BioVision, Mountain View, CA, USA). In vital cell cultures with comparable cell density non-fluorescent resazurin is reduced by dehydrogenases to resorufin emitting fluorescent signals with intensities directly proportional to the oxidative activity of the cells. Toxic compounds interfere with the reduction of resazurin, resulting in decreased signal intensities. The cell culture medium was replaced with medium containing 10% resazurin solution, followed by incubation for 2.5 h at 33°C. Fluorescence was measured at 550/600 nm excitation/emission wavelengths using the Synergy H1 microplate reader (Biotek, Bad Friedrichshall, Germany). Cell culture medium prepared with resazurin alone was used as background control. For data analysis, the signal intensities of Na_2_[PtCl_6_]-treated samples were related to untreated cells as a reference and calculated as percentages (%). According to ISO 10993–5:2009 cell viability of less than 70% relative to the reference was defined as cytotoxic [45].

### Energy-dispersive X-ray analysis (EDAX) of Pt(IV) ions in the HEI-OC1 cell line

Analysis of spatial distribution of Pt(IV) ions within the HEI-OC1 cells was enabled by EDAX. For this experiment, 5 x 10^4^ cells were seeded in duplicate in a 12 well cell culture plate (Nunclon, Thermo Fisher Scientific, Kempen, Germany) on round cover slips wrapped with aluminum foil. Following pre-cultivation for 24 h cells were exposed to 8–12 ng/µL Na_2_[PtCl_6_] for further 48 h as described above. Additionally, cell culture assays without treatment with Na_2_[PtCl_6_] served as control. Following fixation of the cells with 2,5% glutardialdehyde (Polysciences, Warrington, PA, USA) in 0.1 M sodium cacodylate (Th. Geyer, Hamburg, Germany) the samples were dehydrated in graded ethanol (Baker, Phillipsburg, NJ, USA) and dried by critical point drying (EM CPD300, Leica Microsystems GmbH, Wetzlar, Germany). By using the Zeiss Crossbeam 540 (Carl Zeiss Microscopy Deutschland GmbH, Oberkochen, Germany) all relevant electronvolt peaks of duplicates were determined and depicted color-codedly in two dimensions of the culture.

### HEI-OC1 cell cultivation and preparation for patch clamp recordings

For patch-clamp assays, 4,000 HEI-OC1 cells per well were seeded for Na_2_[PtCl_6_] treatment, while 5,000 cells per well served as untreated controls. Control and treated cells were seeded in separate wells of 96-well culture plates (8–10 wells per condition) and pre-cultivated for 24 h. The culture medium of the experimental wells was then replaced with medium containing 8–12 ng/µL Na_2_[PtCl_6_], whereas control wells received fresh medium without compound. Cells were further cultivated for 24 h or 48 h. After 24 h, supernatants were removed and cells were detached using 30–40 µL of 0.05%/0.02% trypsin/EDTA solution per well for 3 min at 33°C and 10% CO_2_. The enzymatic reaction was stopped by adding 200 µL high glucose DMEM supplemented with 10% FCS to each well. After dissociation, the cell suspensions from each well were pooled in a 2 mL Eppendorf tube for centrifugation at 800 rpm and room temperature for 4 min. The supernatant was discarded and the cell pellet was resuspended in 2 mL Leibovitz medium (Gibco/Thermo Fisher Scientific) without FCS, centrifuged as described above, and finally dissolved in 1 mL Leibovitz medium. For analysis, the cell suspension was transferred to the lid of a 35 mm Petri dish and filled up to 4 mL with Leibovitz medium. HEI-OC1 cells treated with Pt were processed in parallel with the corresponding reference protocol.

### Electrophysiology

Whole-cell patch-clamp experiments in the current-clamp configuration were performed on cultured HEI-OC1 cells using patch pipettes, as previously described [46]. Briefly, whole-cell membrane potential recordings were conducted at room temperature (23 ± 2°C) employing an EPC 10 USB double patch-clamp amplifier (HEKA Elektronik Dr. Schulze GmbH, Lambrecht, Germany), with data acquisition controlled via PatchMaster software. Resting membrane potentials (RMPs) were determined in current-clamp mode with zero injected current (*I* = 0) over a 10-s recording period. The whole-cell membrane capacitance was measured using the transient compensation (C-slow) protocol implemented in PatchMaster software. Recorded signals were amplified, low-pass filtered with a 4-pole Bessel filter at a cutoff frequency of 2.9 kHz, digitized at 10 kHz, and stored for subsequent offline analysis.

Patch pipettes were fabricated from borosilicate glass capillaries (GB150F-10; Scientific Products GmbH, Germany) using a P-97 microelectrode puller (Sutter Instruments, Novato, CA, USA), with tip fire-polishing performed via a Microforge (MF-830; Narishige, Japan). For membrane potential recordings, the extracellular bath solution contained (in mM) 142 NaCl, 5 KCl, 2 MgCl_2_, 1.5 CaCl_2_, 5.6 D-glucose, and 10 HEPES, with pH adjusted to 7.4 using NaOH and osmolarity set to 304 mOsmol/L by D-glucose. Patch pipettes were filled with an intracellular solution comprising (in mM) 148 KCl, 2 MgCl_2_, 0.5 CaCl_2_, 1 EGTA, and 10 HEPES, with pH adjusted to 7.4 using KOH and osmolarity adjusted to 298 mOsmol/L with D-glucose; pipette resistances ranged from 3 to 6 MΩ.

Compensation for pipette capacitance, membrane capacitance, and series resistance was achieved using the internal circuitry of the patch-clamp amplifier. All recorded membrane potential values were corrected online during data acquisition for the experimentally determined liquid junction potential (LJP), which was approximately +4.5 mV (bath relative to pipette), according to the method described by Neher *et al*. [47].

### Data analysis and statistics

All data acquired from the relative quantification of the oxidative activity of the HEI-OC1 cell culture assays are presented as mean ± standard deviation (SD) of N = 3 independent experiments, each performed in triplicate. Nonparametric two-way analysis of variance (ANOVA) and Tukey’s multiple comparisons test with a threshold of *p* < 0.05 for significance were used for statistical analysis (GraphPad Prism 7.04). Electrophysiological data were analyzed using SigmaPlot software (version 15.0; Systat Software, San Jose, CA, USA). All electrophysiological measurements reported in this study are presented as mean values ± standard error of the mean (S.E.M). Data normality was assessed using the Shapiro-Wilk test. Statistical significance was determined by one-way ANOVA or Student’s *t*-*t*est, with a threshold of *p* < 0.05. Graphical representations were generated using SigmaPlot and OriginPro software (OriginLab Corporation, Northampton, MA, USA), as appropriate.

## Results

**Fig 1:**
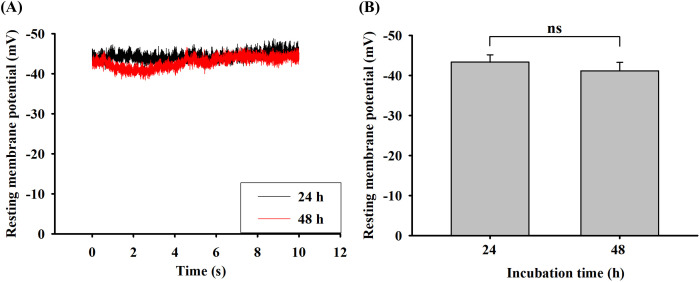
Resting membrane potentials of untreated control HEI-OC1 cells at 24 h and 48h. (A) Representative whole-cell current-clamp traces from untreated HEI-OC1 control cells, showing stable resting membrane potentials. (B) Summary data showing the mean resting potential recorded in current-clamp mode in untreated control HEI-OC1 cells at 24 h (*n* = 18) and 48 h (*n* = 14) of incubation. Results are expressed as mean ± S.E.M. “ns” indicates not significant (*p* > 0.05).

In this study, we employed whole-cell patch-clamp recordings (current-clamp mode, 0 pA holding current) to investigate the electrophysiological effects of sodium hexachloroplatinate (IV) (Na_2_[PtCl_6_]) on cultured HEI-OC1 cells. Under ionic conditions approximating the physiological milieu, LJP-corrected RMPs of untreated control cells remained stable at –43.3 ± 1.9 mV (*n* = 18) and –41.2 ± 2.2 mV (*n* = 14) after 24 and 48 h of incubation, respectively (Fig 1). Mean membrane capacitance values of control HEI-OC1 cells were 11.1 ± 1.7 pF (24 h, *n* = 18) and 14.1 ± 3.1 pF (48 h, *n* = 14). Whole-cell membrane capacitance values obtained from untreated and Na_2_[PtCl_6_]-treated HEI-OC1 cells are summarized in S1 Data. Although slight variations in capacitance were observed among the experimental groups, no statistically significant differences were detected following Na_2_[PtCl_6_] exposure at either 24 h or 48 h.

### Na_2_[PtCl_6_] depolarizes HEI-OC1 cells in a concentration- and time-dependent manner

**Fig 2:**
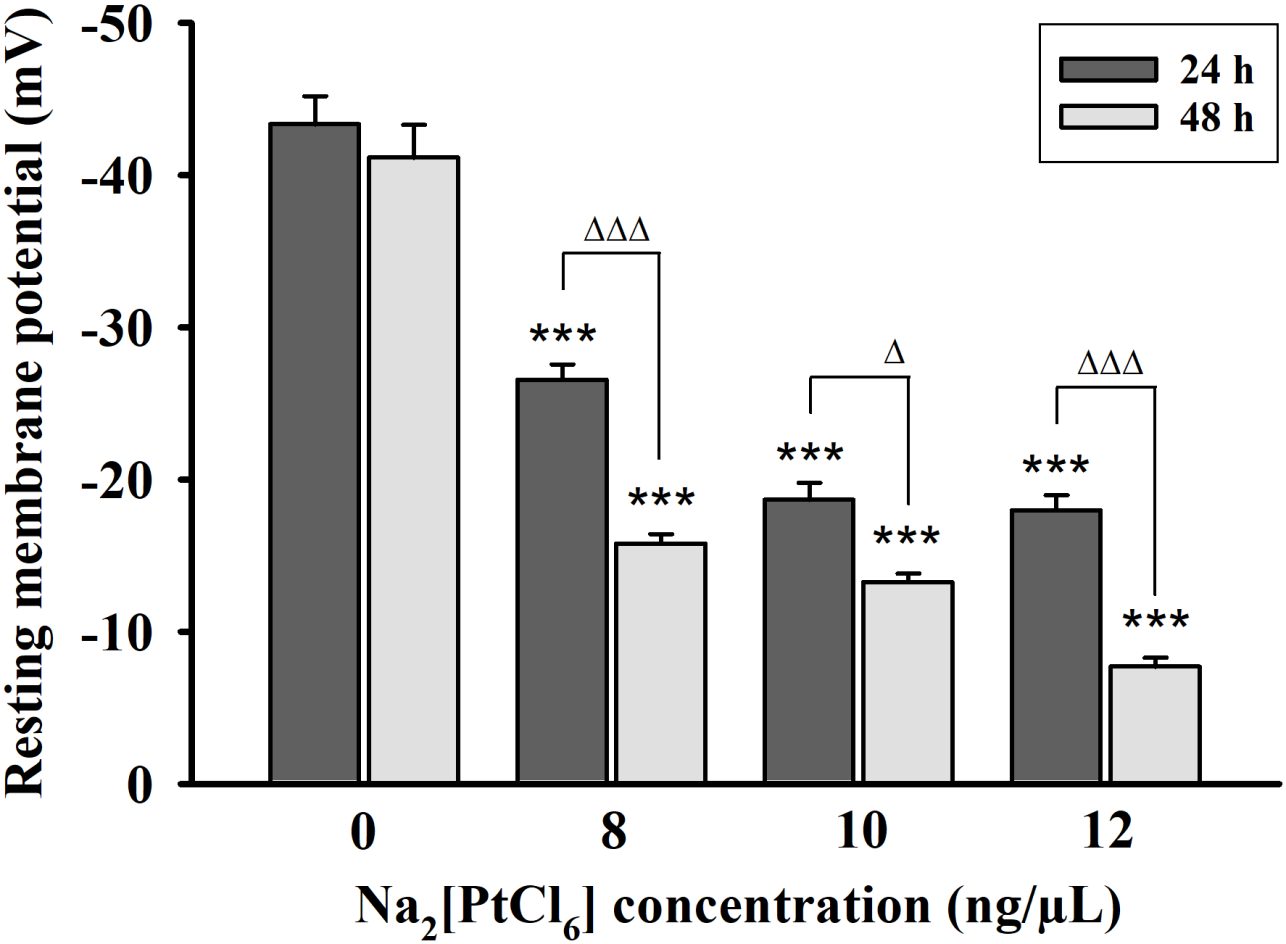
Sodium hexachloroplatinate (IV) (Na_2_[PtCl_6_]) induces concentration- and time-dependent depolarization of HEI-OC1 cells. Summary data illustrating the mean resting potential recorded in current-clamp mode from untreated control HEI-OC1 cells (see Fig 1B.) and cells exposed to 8, 10, and 12 ng/µL Na_2_[PtCl_6_] for 24 h and 48 h. Data are presented as mean ± S.E.M. ^***^, *p* < 0.001 *versus* untreated control at the corresponding time point (comparison among concentrations). ^Δ^, *p* < 0.05 and ^ΔΔΔ^, *p* < 0.001 *versus* the respective 24 h group at the same concentration (time-dependent comparison).

Exposure to Na_2_[PtCl_6_] resulted in a significant, concentration-dependent depolarization of HEI-OC1 cells (Fig 2). After 24 h of treatment with 8, 10 and 12 ng/μL Na_2_[PtCl_6_], the RMPs were depolarized to –26.6 ± 1.0 mV (*n* = 17), –18.7 ± 1.1 mV (*n* = 18), and –18.0 ± 1.0 mV (*n* = 11), respectively. Prolonged exposure (48 h) further enhanced depolarization, with RMPs recorded of –15.8 ± 0.6 mV (*n* = 18), –13.2 ± 0.6 mV (*n* = 19), and –7.7 ± 0.6 mV (*n* = 11) for the corresponding concentrations.

Direct comparison of 24 h and 48 h time points revealed stable membrane potentials in untreated control cells (–43.3 ± 1.9 mV and –41.2 ± 2.2 mV, see also Fig 1B, respectively), whereas Na_2_[PtCl_6_]-treated cells exhibited progressively greater depolarization at all tested concentrations. Statistical analysis confirmed significant differences between corresponding treatment groups across time, indicating a cumulative effect of exposure. Together, these findings demonstrate that Na_2_[PtCl_6_] induces progressive membrane depolarization in HEI-OC1 cells in a concentration- and time-dependent manner and support membrane depolarization as a critical event associated with Na_2_[PtCl_6_]-mediated cytotoxicity.

### Na_2_[PtCl_6_]- induced membrane depolarization correlates with cytotoxicity in HEI-OC1

**Fig 3:**
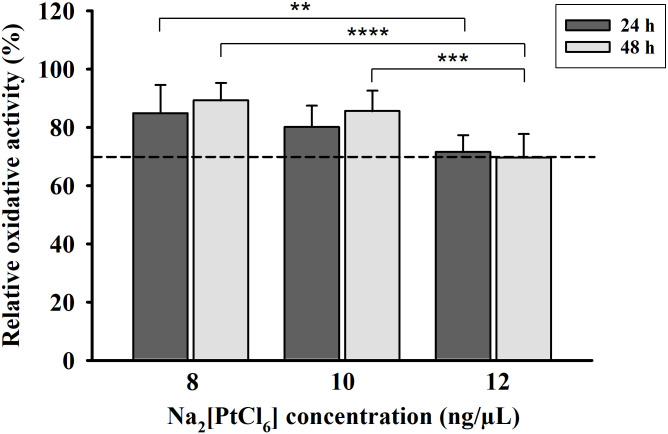
Determination of the relative oxidative activity of HEI-OC1 cells in the presence of 8-12 ng/µL Na_2_[PtCl_6_] for 24 h and 48 h each. The dashed line represents the cytotoxicity limit (70%). Each data point (N = 3 independent experiments in triplicate) is presented as the mean ± SD. Statistical significance is indicated as **p < 0.01, ***p < 0.001 and ****p < 0.0001.

Concurrent with the observed electrophysiological changes, the data of the relative quantification of the oxidative activities of the HEI-OC1 cells revealed an approximately linear concentration dependent decrease of the cell viability: Whereas exposure of 8 ng/μL (84.87 ± 9.74%, 24 h; 89.25 ± 6.06%, 48 h) and 10 ng/µL (80.12 ± 7.36%, 24 h; 85.08 ± 6.75%, 48 h) Na_2_[PtCl_6_] induced moderate cellular changes, supplementation with 12 ng/µL Na_2_[PtCl_6_] resulted in a significant loss of mitochondrial activity (71.57 ± 5.79%, 24 h; 69.59 ± 8.16%, 48 h). As shown in figure 3, Na_2_[PtCl_6_] concentrations of 12 ng/μL and above fulfilled the criteria of ISO10993–5:2009, triggering cell death mechanisms in at least 30% of HEI-OC1 cells relative to the untreated control group. Interestingly, regarding the time-dependent cell stress induction our data did not reflect the electrophysiological changes measured 24 h and 48 h following Na_2_[PtCl_6_] supplementation. The extent of Pt initiated reduction of the oxidative activities in the HEI-OC1 cell culture assays determined after 24 h did not significantly differ from those after cultivation for 48 h of (Fig 3). Notably, while metabolic activity measured by the resazurin assay remained relatively stable between 24 h and 48 h, electrophysiological recordings revealed progressive membrane depolarization over the same time period. This indicates that membrane potential changes represent an earlier functional indicator of Na_2_[PtCl_6_]-induced cellular stress compared to the metabolic cellular activity measurements, suggesting a temporal dissociation between membrane potential changes and metabolic readouts.

### EDAX analysis of the presence and spatial distribution of the [PtCl_6_]^2-^ ions within the HEI-OC1 cells

**Fig 4:**
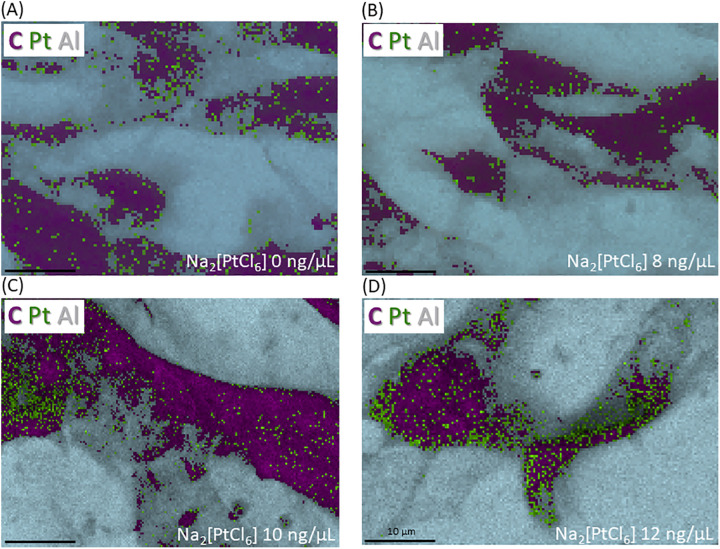
Representative EDAX images of the concentration-dependent distribution of the elements carbon and platinum (Pt) on the aluminum surface. After 48 h incubation period HEI-OC1 cells (depicted by the distribution of carbon in violet dots of different intensity) were cultured on aluminum (in light grey) in presence of the control (**A**), 8 ng/µL (**B**), 10 ng/µL (**C**) and 12 ng/µL (**D**) Na_2_[PtCl_6_], respectively. In addition to single unspecific green dots Pt accumulated in the perinuclear cytoplasm of the adherent ramified HEI-OC1 cells at 10 ng/µL (**C**) and 12 ng/µL (**D**) Na_2_[PtCl_6_] in the culture medium.

EDAX images visualized the presence of aluminum of the whole culture plates and carbon in places of the adherent HEI-OC1 cells connected to each other by ramified thin processes. Importantly, the analysis of platinum revealed unspecific signals in the control group as well the assays exposed to 8 ng/µL Na_2_[PtCl_6_], whereas higher Na_2_[PtCl_6_] concentrations at 10 ng/µL and 12 ng/µL resulted in distinctive accumulation of Pt(IV) in the cytoplasm around the nucleus representing ultrastructurally the region of accumulated mitochondria and the endosomal-lysosomal compartment (Fig 4).

## Discussion

The present study explored the effects of the highly soluble complex Na_2_[PtCl_6_] on membrane potential and mitochondrial oxidative activity of an inner ear related cell line, HEI-OC1, for the first time. Whole-cell patch clamp recordings and relative quantification of oxidative activity were used to evaluate the impact of the Pt(IV) compound in the HEI-OC1 cells at concentrations of 8–12 ng/µL and 24 h and 48 h exposure. Our findings demonstrated a significant depolarization of the RMP in a concentration- and time-dependent manner following Na_2_[PtCl_6_] exposure to the HEI-OC1 cells. Moreover, it could be shown that Na_2_[PtCl_6_] concentrations from 12 ng/µL up induced cell death as revealed by a decrease of the relative oxidative activity.

Whole-cell membrane capacitance (Cslow) was recorded as an indirect indicator of membrane surface area to assess whether the observed electrophysiological alterations were associated with gross changes in cell morphology. Although slight variations in capacitance values were observed among the experimental groups, no statistically significant differences were detected following Na_2_[PtCl_6_] exposure. These findings suggest that the pronounced depolarization of HEI-OC1 cells cannot be explained solely by alterations in membrane surface area or cell size. Rather, the observed changes in resting membrane potential are more likely to reflect functional disturbances of the mechanisms governing membrane polarization, including alterations in ion channel activity and/or ionic homeostasis.

For the assessment of metabolic activity, the non-fluorescent and cell permeable resazurin represents a well characterized biosensor: Following internalization into the cytosol by the target cells resazurin is reduced to resorufin primarily by mitochondrial enzymes, of which fluorescence intensities are measured following release into the cell culture medium [48]. Thus, the reduction capacity of mitochondrial enzymes reflects the metabolic rate and vitality of the cells.

As described in previous studies, the RMP represented a fundamental physiological characteristic of both excitable and non-excitable cells, including auditory hair cells and supporting cells, by preserving ionic gradients critical for normal cellular function and membrane integrity [49–51]. Depolarization leads to disturbance of membrane ion channel functions and alteration of ion permeability resulting in aberrant activation of voltage-gated channels. In particular, disruption of K⁺ channel activity responsible for maintaining the negative RMP may increase cation influx, including Ca^2+^ and Na⁺ [52,53].

By the results of this study the question arises by which molecular pathways Na_2_[PtCl_6_] induce cellular depolarization and an increase of the RMP. First, Na_2_[PtCl_6_] must be internalized by target cells to be able to trigger cytotoxic mechanisms. As it could be shown by earlier reports internalization of antineoplastic Pt compounds as like cisplatin into the HEI-OC1 cells is conducted by the active transfer using membrane transporters such as the copper transporter (Ctr1) and subsequently extruded by the copper-transporting ATPases ATP7A and ATP7B [54–56]. However, passive diffusion of cisplatin along a chloride gradient has also been reported [57].

In contrast to cisplatin, which represents a square planar molecule surrounded by two amino and chloride ligands in a cis-configuration, six chloride ligands enclosed the Pt core in Na_2_[PtCl_6_] forming a spherical structure with high molecular size. The question is how the molecular structure of hexachloroplatinate may impact its cell penetration and damage potential.

As reported previously, cisplatin is converted into aquo complexes after cellular uptake by substitution of chloride ligands with water molecules, enabling irreversible binding to nuclear and mitochondrial DNA and inducing mitochondrial dysfunction and reactive oxygen species generation [22,57,58]. It was shown that Na_2_[PtCl_6_] can impair mitochondrial activities followed by necroptosis [27]. Our EDAX data demonstrated the accumulation of the element Pt near the nucleus, particularly in the region of assembled mitochondria and the endosomal-lysosomal compartment indicating Na_2_[PtCl_6_] initiated interference of the oxidative respiratory chain to ATP generation and disturbance of the active membrane transporter processes [56–59].

Previous studies have noted that elevated intracellular Ca^2+^ is a common pathway to cell death in sensorineural hearing loss [60,61]. Additionally, K⁺ outflow due to changes in potassium channel activity, as well as increased Na⁺ entry via voltage-gated sodium channels, were shown to be involved in caspase-3 activation and the induction of neuronal apoptosis [62]. However, the baseline expression levels and current densities of voltage-gated sodium, calcium, and potassium channels were not directly assessed in the HEI-OC1 sub-clone used in the present study. Therefore, the involvement of specific voltage-gated ion channels in Na_2_[PtCl_6_]-induced depolarization and cytotoxicity remains speculative and requires further investigation.

Ultrastructural analysis of HEI-OC1 cells revealed Na_2_[PtCl_6_] induced mitochondrial dysfunction resulting in ATP depletion and reduction in dehydrogenases [27].

Consistent with our electrophysiological findings demonstrating concentration- and time-dependent depolarization of HEI-OC1 cells, assessment of mitochondrial oxidative activity revealed a concentration-dependent reduction in cell viability, with no significant difference between 24 h and 48 h exposure. HEI-OC1 cells exposed to 14 ng/µL Na_2_[PtCl_6_] revealed mitophagy, indicating activation of intrinsic mitochondrial repair mechanisms. Nevertheless, prolonged exposure led to organelle swelling and loss of membrane integrity, consistent with necroptotic cell death [27].

Together, these results indicate that Na_2_[PtCl_6_] disrupts auditory cell electrophysiology and mitochondrial function in a concentration-dependent manner. The progressive depolarization observed in the absence of major changes in whole-cell membrane capacitance suggests that alterations in ion homeostasis may represent an early functional manifestation of Pt(IV)-induced cellular stress preceding overt metabolic deterioration. Further studies addressing the identity and regulation of the ion channels involved may contribute to a better understanding of the mechanisms underlying Pt-induced ototoxicity.

## Conclusion

Our results demonstrate that the model Pt(IV) complex Na_2_[PtCl_6_], a potential platinum corrosion product relevant to cochlear implants, induces electrophysiological alterations in auditory HEI-OC1 cells in a concentration- and time-dependent manner. These alterations were characterized by depolarization of the resting membrane potential, suggesting a critical cellular mechanism linking Pt(IV) exposure to impaired auditory cell function.

These findings underscore the necessity for improved corrosion-resistant electrode materials, optimization of long-term charge balance and stimulation protocols, and the development of protective coatings to limit platinum ion release.

While HEI-OC1 cells provide a relevant *in vitro* model, validation of these effects *in vivo* within the complex cochlear milieu is required. Future studies should aim to identify the specific ion channel subtypes responsible for Na_2_[PtCl_6_]-induced membrane depolarization at the implant interface. Such insights will be pivotal for enhancing cochlear implant biocompatibility and preserving auditory function.

## Supporting information

S1 Data Minimal dataset. Minimal underlying dataset for all statistical analyses. Individual resting membrane potential (RMP), whole-cell membrane capacitance (Cslow), and resazurin assay measurements used in the statistical analyses presented in the manuscript.
(XLSX)
