# Supplementary material for: Sodium hexachloroplatinate (IV) induces concentration- and time-dependent depolarization and cytotoxicity in HEI-OC1 auditory cells
Source: PLoS One. 2026 Jul 22;21(7):e0354369. doi: 10.1371/journal.pone.0354369 (PMC13390847; doi:10.1371/journal.pone.0354369)
Supplement: Attachment — Submitted filename: Response to Reviewers.docx [file pone.0354369.s003.docx]

**Response to Reviewers**

**Manuscript Title:** Sodium Hexachloroplatinate (IV) Induces Concentration- and Time-Dependent Depolarization and Cytotoxicity in HEI-OC1 Auditory Cells

**Manuscript Number:** PONE-D-26-18759

Dear Dr. Prashanth S. Adarakatti,

We sincerely thank you and the reviewers for the careful evaluation of our manuscript and for the constructive comments and suggestions. We appreciate the opportunity to revise our work. The comments have been invaluable in improving the scientific rigor, clarity, and overall quality of the manuscript.

We have carefully considered all comments and have revised the manuscript accordingly. All changes have been highlighted in the revised version. Below, we provide a detailed point-by-point response to each comment.

**Note**: All line numbers cited below refer to the Revised Manuscript with Track Changes.

**JOURNAL REQUIREMENTS**

**Comment 1:**

Please ensure that your manuscript meets PLOS ONE's style requirements, including those for file naming.

**Response:**

We have thoroughly reviewed the manuscript and revised it to comply fully with PLOS ONE formatting and file-naming requirements.

**Comment 2:**

In the Methods, please ensure you state the commercial source/company that supplied the primary cells used in this study.

**Response:**

The source information for the HEI-OC1 cells has now been added to the Methods section (Lines 114–117).

**Comment 3:**

Please state what role the funders took in the study.

**Response:**

The funding-related text has been removed from the manuscript in accordance with the Plos One editorial requirements. The funding information, including the statement regarding the role of the funders, has been provided in the Funding Statement section of the online submission system and has also been included in the cover letter for completeness.

The funders had no role in study design, data collection and analysis, decision to publish, or preparation of the manuscript.

**Comment 4:**

Please confirm at this time whether or not your submission contains all raw data required to replicate the results of your study.

**Response:**

We confirm that all raw data underlying the findings reported in this manuscript have been provided as Supporting Information (S1 Data). A description of S1 Data has been added to the Supporting Information section of the revised manuscript (Lines 415–420). The Data Availability Statement has been updated accordingly (Line 427).

**ADDITIONAL EDITOR COMMENTS**

**Comment 1:**

Why does the resting membrane potential show progressive, time-dependent depolarization from 24 h to 48 h (Fig 2), while the relative oxidative activity remains completely unchanged between these two time points (Fig 3)?

**Response:**

We thank the Editor for this important observation. The apparent discrepancy between the progressive, time-dependent depolarization of the resting membrane potential (Fig. 2) and the absence of further changes in resazurin-based oxidative activity between 24 h and 48 h (Fig. 3) reflects differences in the biological processes measured and their respective temporal sensitivities.

Whole-cell patch-clamp recordings detect early functional alterations in membrane properties, which are highly sensitive to ionic and electrophysiological disturbances and may precede overt metabolic decline. In contrast, the resazurin assay reflects overall cellular metabolic activity and mitochondrial function, which typically represents a later and more integrated endpoint of cytotoxicity modulated by concurrent repair mechanisms (e.g. mitophagy).

Therefore, the observed time-dependent depolarization between 24 h and 48 h likely reflects progressive electrophysiological impairment, while the metabolic activity reaches an early plateau of reduction after initial exposure and does not show further significant decline within the same time window.

We have clarified this temporal dissociation between electrophysiological dysfunction and metabolic readouts in the revised Results (lines 279–285) and Discussion (lines 393–399) sections.

**Comment 2:**

Since the authors hypothesize that Na_2_[PtCl_6_]-induced depolarization facilitates the activation of voltage-gated channels, what are the baseline expressions or current densities of voltage-gated Na^+^, Ca^2+^, or K^+^ channels in this specific HEI-OC1 sub-clone?

**Response:**

We thank the Editor for this important mechanistic question. The baseline expression and current densities of voltage-gated Na^+^, Ca^2+^, and K^+^ channels were not experimentally determined in the HEI-OC1 sub-clone used in this study. Accordingly, we cannot provide direct measurements addressing this point. We have revised the Discussion (lines 374–379) to explicitly state this limitation and to clarify that the proposed involvement of voltage-gated channels in the observed depolarization remains speculative and is not supported by direct functional evidence in the present work.

**Comment 3:**

The methods state that whole-cell membrane capacitance (Cslow) was recorded to reflect modifications in cell size or surface area, but these data are missing from the results; could the authors provide these values to complement the RMP changes?

**Response:**

We thank the Editor for this helpful suggestion. We have analyzed and included whole-cell membrane capacitance (Cslow) measurements in the Results section of the revised manuscript (Lines 226–231). The corresponding data are provided in the Supporting Information file (S1 Data). Although Cslow was recorded as an indirect indicator of cell surface area, no statistically significant differences were observed between experimental groups. A statement clarifying this has been added to the Discussion (Lines 322–332). These findings suggest that the Na_2_[PtCl_6_]-induced membrane depolarization is unlikely to be explained by changes in cell size or membrane surface area alone but rather may reflect alterations in ion homeostasis and/or ion channel activity.

**Comment 4:**

Figure 4 labels denote exposure units in "µg/ml" (which equals ng/µL), but the figure caption refers to them as "8, 10, and 12 ng/µL"; please standardize the concentration units across the manuscript and figures for consistency.

**Response:**

We thank the Editor for identifying this inconsistency. The labels in Figure 4 have been corrected, and concentration units have been standardized throughout the manuscript, figures, and figure legends for consistency and clarity. Specifically, the Figure 4 labels were revised to ng/µL to align with the figure caption and the manuscript text.

**Comment 5:**

Given the specific ionic composition of your extracellular bath (142 mM Na^+^) and internal pipette solution (148 mM K^+^), what was the exact experimentally determined liquid junction potential value used for correction?

**Response:**

We appreciate this important methodological question. The liquid junction potential (LJP) was experimentally measured in I = 0 mode as the voltage difference between the pipette and bath solutions. The measured LJP was approximately −4.5 mV (pipette relative to bath) and was corrected online during data acquisition using the Auto V_o_ function in PatchMaster software (HEKA). This methodological detail has now been added to the Electrophysiology subsection of the Materials and Methods section (lines 203–205).

**Comment 6:**

To enhance the manuscript’s impact and appeal to a broader scientific readership, consider incorporating recent references to ensure completeness. These references should be included in the Introduction or other relevant sections as appropriate. Additionally, avoid citing more than five references within a single sentence to maintain clarity and readability.

**Response:**

We thank the Editor for this valuable recommendation. All suggested references were carefully evaluated, and those directly relevant to heavy-metal toxicity, platinum-induced cellular injury, mitochondrial dysfunction, oxidative stress, neuroinflammation, auditory-cell damage, and neuroprotective mechanisms have been incorporated into the Introduction (lines 66–77).

In addition, the citation format was revised throughout the manuscript to improve readability and avoid excessive clustering of references, ensuring compliance with the journal’s guidelines.

**CONCLUSION**

We believe that the revisions have substantially strengthened the manuscript and addressed all concerns raised by the Editor. We are grateful for the thoughtful comments and suggestions, which have significantly improved the quality and clarity of our work.

Thank you for your time and consideration. We hope that the revised manuscript is now suitable for publication in PLOS ONE.

Sincerely,

Hamdy Embark, PhD Corresponding

On behalf of all authors

E-Mail: [Embark.Hamdy@mh-hannover.de](mailto:Embark.Hamdy@mh-hannover.de)
