# Supplementary material for: Sodium hexachloroplatinate (IV) induces concentration- and time-dependent depolarization and cytotoxicity in HEI-OC1 auditory cells
Source: PLoS One. 2026 Jul 22;21(7):e0354369. doi: 10.1371/journal.pone.0354369 (PMC13390847; doi:10.1371/journal.pone.0354369)
Supplement: Peer review history

# Editor report: Decision Letter 0

**DOI:** 10.1371/journal.pone.0354369.r001

**Authors:** Prashanth S. Adarakatti

28 May 2026

PONE-D-26-18759

Sodium Hexachloroplatinate (IV) Induces Concentration- and Time-Dependent Depolarization and Cytotoxicity in HEI-OC1 Auditory Cells

PLOS One

Dear Dr. Embark,

Thank you for submitting your manuscript to PLOS ONE. After careful consideration, we feel that it has merit but does not fully meet PLOS ONE’s publication criteria as it currently stands. Therefore, we invite you to submit a revised version of the manuscript that addresses the points raised during the review process.

Please submit your revised manuscript by Jul 12 2026 11:59PM. If you will need more time than this to complete your revisions, please reply to this message or contact the journal office at plosone@plos.org. When you're ready to submit your revision, log on to https://www.editorialmanager.com/pone/ and select the 'Submissions Needing Revision' folder to locate your manuscript file.

Please include the following items when submitting your revised manuscript:

- A letter that responds to each point raised by the academic editor and reviewer(s). You should upload this letter as a separate file labeled 'Response to Reviewers'.
- A marked-up copy of your manuscript that highlights changes made to the original version. You should upload this as a separate file labeled 'Revised Manuscript with Track Changes'.
- An unmarked version of your revised paper without tracked changes. You should upload this as a separate file labeled 'Manuscript'.

If you would like to make changes to your financial disclosure, please include your updated statement in your cover letter. Guidelines for resubmitting your figure files are available below the reviewer comments at the end of this letter.

If applicable, we recommend that you deposit your laboratory protocols in protocols.io to enhance the reproducibility of your results. Protocols.io assigns your protocol its own identifier (DOI) so that it can be cited independently in the future. For instructions see: https://journals.plos.org/plosone/s/submission-guidelines#loc-laboratory-protocols. Additionally, PLOS ONE offers an option for publishing peer-reviewed Lab Protocol articles, which describe protocols hosted on protocols.io. Read more information on sharing protocols at https://plos.org/protocols?utm_medium=editorial-email&utm_source=authorletters&utm_campaign=protocols.

As the corresponding author, your ORCID iD is verified in the submission system and will appear in the published article. PLOS supports the use of ORCID, and we encourage all coauthors to register for an ORCID iD and use it as well. Please encourage your coauthors to verify their ORCID iD within the submission system before final acceptance, as unverified ORCID iDs will not appear in the published article. *Only*  the individual author can complete the verification step; PLOS staff *cannot*  verify ORCID iDs on behalf of authors.

We look forward to receiving your revised manuscript.

Kind regards,

Prashanth S. Adarakatti

Academic Editor

PLOS One

Journal Requirements:

When submitting your revision, we need you to address these additional requirements.

1. Please ensure that your manuscript meets PLOS ONE's style requirements, including those for file naming. The PLOS ONE style templates can be found at

https://journals.plos.org/plosone/s/file?id=wjVg/PLOSOne_formatting_sample_main_body.pdf and

https://journals.plos.org/plosone/s/file?id=ba62/PLOSOne_formatting_sample_title_authors_affiliations.pdf

2. In the Methods, please ensure you state the commercial source/company that supplied the primary cells used in this study.

3. Thank you for stating the following financial disclosure:

“This study is funded by the Deutsche Forschungsgemeinschaft (DFG, German Research Foundation) – SFB/TRR 298 SIIRI – Project ID 426335750, as well as under Germany’s Excellence Strategy – EXC 2177/2 – Project ID 390895286.”

Please state what role the funders took in the study.  If the funders had no role, please state: "The funders had no role in study design, data collection and analysis, decision to publish, or preparation of the manuscript."

If this statement is not correct you must amend it as needed.

Please include this amended Role of Funder statement in your cover letter; we will change the online submission form on your behalf.

4. We note that your Data Availability Statement is currently as follows: “All relevant data are within the manuscript and are fully available without restriction”

Please confirm at this time whether or not your submission contains all raw data required to replicate the results of your study. Authors must share the “minimal data set” for their submission. PLOS defines the minimal data set to consist of the data required to replicate all study findings reported in the article, as well as related metadata and methods (https://journals.plos.org/plosone/s/data-availability#loc-minimal-data-set-definition).

For example, authors should submit the following data:

- The values behind the means, standard deviations and other measures reported;

- The values used to build graphs;

- The points extracted from images for analysis.

Authors do not need to submit their entire data set if only a portion of the data was used in the reported study.

If your submission does not contain these data, please either upload them as Supporting Information files or deposit them to a stable, public repository and provide us with the relevant URLs, DOIs, or accession numbers. For a list of recommended repositories, please see https://journals.plos.org/plosone/s/recommended-repositories.

If there are ethical or legal restrictions on sharing a de-identified data set, please explain them in detail (e.g., data contain potentially sensitive information, data are owned by a third-party organization, etc.) and who has imposed them (e.g., an ethics committee). Please also provide contact information for a data access committee, ethics committee, or other institutional body to which data requests may be sent. If data are owned by a third party, please indicate how others may request data access.

5. If the reviewer comments include a recommendation to cite specific previously published works, please review and evaluate these publications to determine whether they are relevant and should be cited. There is no requirement to cite these works unless the editor has indicated otherwise.

Additional Editor Comments:

The present work describes the “Sodium Hexachloroplatinate (IV) Induces Concentration- and Time-Dependent Depolarization and Cytotoxicity in HEI-OC1 Auditory Cells”. The proposed work can be accepted only after answering few queries.

Here are my remarks:

1) Why does the resting membrane potential show progressive, time-dependent depolarization from 24 h to 48 h (Fig 2), while the relative oxidative activity remains completely unchanged between these two time points (Fig 3)?

2) Since the authors hypothesize that Na2[PtCl6]-induced depolarization facilitates the activation of voltage-gated channels, what are the baseline expressions or current densities of voltage-gated Na+, Ca2+, or K+ channels in this specific HEI-OC1 sub-clone?

3) The methods state that whole-cell membrane capacitance (Cslow) was recorded to reflect modifications in cell size or surface area, but these data are missing from the results; could the authors provide these values to complement the RMP changes?

4) Figure 4 labels denote exposure units in "μg/ml" (which equals ng/μL), but the figure caption refers to them as "8, 10, and 12 ng/μL"; please standardize the concentration units across the manuscript and figures for consistency.

5) Given the specific ionic composition of your extracellular bath (142 mM Na+) and internal pipette solution (148 mM K+), what was the exact experimentally determined liquid junction potential value used for correction?

6) To enhance the manuscript's impact and appeal to a broader scientific readership, consider incorporating recent references to ensure completeness. These references should be included in the Introduction or other relevant sections as appropriate. Additionally, avoid citing more than five references within a single sentence to maintain clarity and readability.

Authors are encouraged to write the sentences below in the introduction section, incorporating the citations to enhance broader appeal and readability.

Heavy metal toxicity pathways, such cadmium-induced c-Myc changes [1] and the mitochondrial apoptotic profiles monitored by targeted triphenylphosphonium micelles [2], are mirrored in the metabolic and transcriptional disruption caused by Na2[PtCl6] in HEI-OC1 cells. Ionic permeability is changed as a result of the membrane depolarization, and important signaling networks such as CREB regulation through zinc complexes [3] or NF-κB activation observed in chemotherapy cardiotoxicity [4] are downstream of this. Additionally, assessing the neurotoxicity of this platinum compound mirrors the ion-balance issues seen in models of alumina nanoparticles [5]. Central microglial auditory control [6], tissue repair mechanisms like the miR-760-3p/GPR17 axis [7], and synaptic modulators like pexidartinib [8] must all be taken into consideration in more comprehensive clinical remediation. Mapping sex-specific bioavailability and pharmacokinetics [9] is ultimately necessary to assure localized otoprotection when switching from Pt(IV)-induced necroptosis to protective interventions, akin to chrysotoxine-mediated ferroptosis regulation [10].

1) doi: https://doi.org/10.1016/j.taap.2021.115536

2) doi: 10.2147/IJN.S219670

3) doi: https://doi.org/10.1002/cjoc.202200406

4) doi: https://doi.org/10.1016/j.biopha.2020.110525

5) doi: https://doi.org/10.1016/j.fct.2021.112587

6) doi: https://doi.org/10.1016/j.ejphar.2024.176759

7) doi: https://doi.org/10.1016/j.jep.2024.118126

8) doi: https://doi.org/10.1016/j.intimp.2022.109436

9) doi: https://doi.org/10.3390/ph17081053

10) doi: https://doi.org/10.1016/j.jep.2024.119126

[Note: HTML markup is below. Please do not edit.]

[NOTE: If reviewer comments were submitted as an attachment file, they will be attached to this email and accessible via the submission site. Please log into your account, locate the manuscript record, and check for the action link "View Attachments". If this link does not appear, there are no attachment files.]

To ensure your figures meet our technical requirements, please review our figure guidelines: https://journals.plos.org/plosone/s/figures

You may also use PLOS’s free figure tool, NAAS, to help you prepare publication quality figures: https://journals.plos.org/plosone/s/figures#loc-tools-for-figure-preparation.

NAAS will assess whether your figures meet our technical requirements by comparing each figure against our figure specifications.

# Author comment: Author response to Decision Letter 1

**DOI:** 10.1371/journal.pone.0354369.r002

19 Jun 2026

Response to Reviewers

Manuscript Title: Sodium Hexachloroplatinate (IV) Induces Concentration- and Time-Dependent Depolarization and Cytotoxicity in HEI-OC1 Auditory Cells

Manuscript Number: PONE-D-26-18759

Dear Dr. Prashanth S. Adarakatti,

We sincerely thank you and the reviewers for the careful evaluation of our manuscript and for the constructive comments and suggestions. We appreciate the opportunity to revise our work. The comments have been invaluable in improving the scientific rigor, clarity, and overall quality of the manuscript.

We have carefully considered all comments and have revised the manuscript accordingly. All changes have been highlighted in the revised version. Below, we provide a detailed point-by-point response to each comment.

Note: All line numbers cited below refer to the Revised Manuscript with Track Changes.

JOURNAL REQUIREMENTS

Comment 1:

Please ensure that your manuscript meets PLOS ONE's style requirements, including those for file naming.

Response:

We have thoroughly reviewed the manuscript and revised it to comply fully with PLOS ONE formatting and file-naming requirements.

Comment 2:

In the Methods, please ensure you state the commercial source/company that supplied the primary cells used in this study.

Response:

The source information for the HEI-OC1 cells has now been added to the Methods section (Lines 114–117).

Comment 3:

Please state what role the funders took in the study.

Response:

The funding-related text has been removed from the manuscript in accordance with the Plos One editorial requirements. The funding information, including the statement regarding the role of the funders, has been provided in the Funding Statement section of the online submission system and has also been included in the cover letter for completeness.

The funders had no role in study design, data collection and analysis, decision to publish, or preparation of the manuscript.

Comment 4:

Please confirm at this time whether or not your submission contains all raw data required to replicate the results of your study.

Response:

We confirm that all raw data underlying the findings reported in this manuscript have been provided as Supporting Information (S1 Data). A description of S1 Data has been added to the Supporting Information section of the revised manuscript (Lines 415–420). The Data Availability Statement has been updated accordingly (Line 427).

ADDITIONAL EDITOR COMMENTS

Comment 1:

Why does the resting membrane potential show progressive, time-dependent depolarization from 24 h to 48 h (Fig 2), while the relative oxidative activity remains completely unchanged between these two time points (Fig 3)?

Response:

We thank the Editor for this important observation. The apparent discrepancy between the progressive, time-dependent depolarization of the resting membrane potential (Fig. 2) and the absence of further changes in resazurin-based oxidative activity between 24 h and 48 h (Fig. 3) reflects differences in the biological processes measured and their respective temporal sensitivities.

Whole-cell patch-clamp recordings detect early functional alterations in membrane properties, which are highly sensitive to ionic and electrophysiological disturbances and may precede overt metabolic decline. In contrast, the resazurin assay reflects overall cellular metabolic activity and mitochondrial function, which typically represents a later and more integrated endpoint of cytotoxicity modulated by concurrent repair mechanisms (e.g. mitophagy).

Therefore, the observed time-dependent depolarization between 24 h and 48 h likely reflects progressive electrophysiological impairment, while the metabolic activity reaches an early plateau of reduction after initial exposure and does not show further significant decline within the same time window.

We have clarified this temporal dissociation between electrophysiological dysfunction and metabolic readouts in the revised Results (lines 279–285) and Discussion (lines 393–399) sections.

Comment 2:

Since the authors hypothesize that Na2[PtCl6]-induced depolarization facilitates the activation of voltage-gated channels, what are the baseline expressions or current densities of voltage-gated Na+, Ca2+, or K+ channels in this specific HEI-OC1 sub-clone?

Response:

We thank the Editor for this important mechanistic question. The baseline expression and current densities of voltage-gated Na+, Ca2+, and K+ channels were not experimentally determined in the HEI-OC1 sub-clone used in this study. Accordingly, we cannot provide direct measurements addressing this point. We have revised the Discussion (lines 374–379) to explicitly state this limitation and to clarify that the proposed involvement of voltage-gated channels in the observed depolarization remains speculative and is not supported by direct functional evidence in the present work.

Comment 3:

The methods state that whole-cell membrane capacitance (Cslow) was recorded to reflect modifications in cell size or surface area, but these data are missing from the results; could the authors provide these values to complement the RMP changes?

Response:

We thank the Editor for this helpful suggestion. We have analyzed and included whole-cell membrane capacitance (Cslow) measurements in the Results section of the revised manuscript (Lines 226–231). The corresponding data are provided in the Supporting Information file (S1 Data). Although Cslow was recorded as an indirect indicator of cell surface area, no statistically significant differences were observed between experimental groups. A statement clarifying this has been added to the Discussion (Lines 322–332). These findings suggest that the Na2[PtCl6]-induced membrane depolarization is unlikely to be explained by changes in cell size or membrane surface area alone but rather may reflect alterations in ion homeostasis and/or ion channel activity.

Comment 4:

Figure 4 labels denote exposure units in "µg/ml" (which equals ng/µL), but the figure caption refers to them as "8, 10, and 12 ng/µL"; please standardize the concentration units across the manuscript and figures for consistency.

Response:

We thank the Editor for identifying this inconsistency. The labels in Figure 4 have been corrected, and concentration units have been standardized throughout the manuscript, figures, and figure legends for consistency and clarity. Specifically, the Figure 4 labels were revised to ng/µL to align with the figure caption and the manuscript text.

Comment 5:

Given the specific ionic composition of your extracellular bath (142 mM Na+) and internal pipette solution (148 mM K+), what was the exact experimentally determined liquid junction potential value used for correction?

Response:

We appreciate this important methodological question. The liquid junction potential (LJP) was experimentally measured in I = 0 mode as the voltage difference between the pipette and bath solutions. The measured LJP was approximately −4.5 mV (pipette relative to bath) and was corrected online during data acquisition using the Auto Vo function in PatchMaster software (HEKA). This methodological detail has now been added to the Electrophysiology subsection of the Materials and Methods section (lines 203–205).

Comment 6:

To enhance the manuscript’s impact and appeal to a broader scientific readership, consider incorporating recent references to ensure completeness. These references should be included in the Introduction or other relevant sections as appropriate. Additionally, avoid citing more than five references within a single sentence to maintain clarity and readability.

Response:

We thank the Editor for this valuable recommendation. All suggested references were carefully evaluated, and those directly relevant to heavy-metal toxicity, platinum-induced cellular injury, mitochondrial dysfunction, oxidative stress, neuroinflammation, auditory-cell damage, and neuroprotective mechanisms have been incorporated into the Introduction (lines 66–77).

In addition, the citation format was revised throughout the manuscript to improve readability and avoid excessive clustering of references, ensuring compliance with the journal’s guidelines.

CONCLUSION

We believe that the revisions have substantially strengthened the manuscript and addressed all concerns raised by the Editor. We are grateful for the thoughtful comments and suggestions, which have significantly improved the quality and clarity of our work.

Thank you for your time and consideration. We hope that the revised manuscript is now suitable for publication in PLOS ONE.

Sincerely,

Hamdy Embark, PhD Corresponding

On behalf of all authors

E-Mail: Embark.Hamdy@mh-hannover.de

Attachment Submitted filename: Response to Reviewers.docx

# Editor report: Decision Letter 1

**DOI:** 10.1371/journal.pone.0354369.r003

**Authors:** Prashanth S. Adarakatti

8 Jul 2026

Sodium Hexachloroplatinate (IV) Induces Concentration- and Time-Dependent Depolarization and Cytotoxicity in HEI-OC1 Auditory Cells

PONE-D-26-18759R1

Dear Embark,

We’re pleased to inform you that your manuscript has been judged scientifically suitable for publication and will be formally accepted for publication once it meets all outstanding technical requirements.

Within one week, you’ll receive an e-mail detailing the required amendments. When these have been addressed, you’ll receive a formal acceptance letter and your manuscript will be scheduled for publication.

An invoice will be generated when your article is formally accepted. Please note, if your institution has a publishing partnership with PLOS and your article meets the relevant criteria, all or part of your publication costs will be covered. Please make sure your user information is up-to-date by logging into Editorial Manager at Editorial Manager® and clicking the ‘Update My Information' link at the top of the page. For questions related to billing, please contact billing support.

If your institution or institutions have a press office, please notify them about your upcoming paper to help maximize its impact. If they’ll be preparing press materials, please inform our press team as soon as possible -- no later than 48 hours after receiving the formal acceptance. Your manuscript will remain under strict press embargo until 2 pm Eastern Time on the date of publication. For more information, please contact onepress@plos.org.

Kind regards,

Prashanth S. Adarakatti

Academic Editor

PLOS One

Additional Editor Comments (optional):

Accept.

Reviewers' comments:

# Editor report: Acceptance letter

**DOI:** 10.1371/journal.pone.0354369.r004

**Authors:** Prashanth S. Adarakatti

PONE-D-26-18759R1

PLOS One

Dear Dr. Embark,

I'm pleased to inform you that your manuscript has been deemed suitable for publication in PLOS One. Congratulations! Your manuscript is now being handed over to our production team.

At this stage, our production department will prepare your paper for publication. This includes ensuring the following:

* All references, tables, and figures are properly cited

* All relevant supporting information is included in the manuscript submission,

* There are no issues that prevent the paper from being properly typeset

You will receive further instructions from the production team, including instructions on how to review your proof when it is ready. Please keep in mind that we are working through a large volume of accepted articles, so please give us a few days to review your paper and let you know the next and final steps.

Lastly, if your institution or institutions have a press office, please let them know about your upcoming paper now to help maximize its impact. If they'll be preparing press materials, please inform our press team within the next 48 hours. Your manuscript will remain under strict press embargo until 2 pm Eastern Time on the date of publication. For more information, please contact onepress@plos.org.

You will receive an invoice from PLOS for your publication fee after your manuscript has reached the completed accept phase. If you receive an email requesting payment before acceptance or for any other service, this may be a phishing scheme. Learn how to identify phishing emails and protect your accounts at https://explore.plos.org/phishing.

If we can help with anything else, please email us at customercare@plos.org.

Thank you for submitting your work to PLOS ONE and supporting open access.

Kind regards,

PLOS ONE Editorial Office Staff

on behalf of

Dr. Prashanth S. Adarakatti

Academic Editor

PLOS One
